# Meat spoilage by bacteria: Influencing factors, volatile compounds, and organoleptic alterations

**DOI:** 10.1007/s44463-025-00025-w

**Published:** 2026-03-03

**Authors:** Joohyun Kang, Byung Hee Kim, Yohan Yoon

**Affiliations:** 1 Risk Analysis Research Center, Sookmyung Women’s University, 04310 Seoul, Korea; 2 Department of Food and Nutrition, Sookmyung Women’s University, 04310 Seoul, Korea

**Keywords:** Meat spoilage bacteria, Spoilage modes, Volatile compounds, Organoleptic alterations

## Abstract

This review provides an understanding of spoilage factors of meat, the spoilage bacteria, the spoilage mode of major spoilage bacteria, and alterations due to spoilage. pH and water activity of meat, packaging atmosphere, and storage temperature affect the growth and activity of spoilage bacteria. The spoilage bacteria cause the production of various compounds. Volatile compounds such as volatile basic nitrogen [ammonia (NH_3_) and amines (RNH_2_)], alcohols (ROH), aldehydes (RCHO), ketones (RCOR’), and sulfur (S) compounds are generated by decomposition from glycogen, proteins and lipids in meat with various enzymatic (decarboxylase, phosphoketolase, alcohol dehydrogenase, lipoxygenase, acetate kinase, etc.) reactions of *Brochothrix thermosphacta*, *Carnobacterium*, Enterobacteriaceae, lactic acid bacteria, *Pseudomonas*, etc. During the spoilage process, some metabolic activities of bacteria lead to organoleptic alterations in meat. Some bacteria form slime, produce odor-causing substances, or cause color changes during meat spoilage. The information reviewed in this paper may help readers better understand meat spoilage by bacteria; other causes responsible for spoilage are not covered in this paper.

## Introduction

Meat spoilage mainly occurs due to decomposition and the formation of metabolites by bacterial growth (Gracias & McKillip, 2004). Spoilage bacteria are important contributors to meat quality deterioration during storage (Doulgeraki et al., 2012). Growth of spoilage bacteria decreases the shelf-life of meat and increases waste, which results in economic losses in the meat industry (Mohareb et al., 2015).

The spoilage bacteria can produce volatile compounds (VCs) such as volatile basic nitrogen [VBN: ammonia (NH_3_) and amines (RNH_2_)], aldehydes (RCHO), alcohols (ROH), esters (RCOOR), ketones (RCOR’), sulfur (S) compounds, and volatile organic acids (VOAs) (Frank et al., 2020; Kakouri & Nychas, 1994; Lambert et al., 1991). The rate and types of spoilage depend on the concentrations of glycogen, lactic acid, nitrogen compounds, and free amino acids present in meat, which are the main precursors of microbial metabolites related to spoilage (Nychas et al., 1998, 2008; Tsigarida & Nychas, 2001). The glycogen and nitrogen compounds produced different VCs depending on the bacterial species and oxygen affinity (Casaburi et al., 2015).

Although oxidation and autolysis in meat play an important role in meat spoilage, they have been widely reviewed by scientists (Dave & Ghaly, 2011; Huff-Lonergan & Lonergan, 2005; Zhang et al., 2013). However, the research results on bacterial meat spoilage (Casaburi et al., 2015; Doulgeraki et al., 2012; Nychas et al., 2008) are scattered and fragmented. To the best of our knowledge, the types of spoilage bacteria, their metabolic modes, the biochemical changes by spoilage bacteria, etc., have not been reviewed comprehensively.

Therefore, this paper reviewed the factors and bacteria that cause meat spoilage, the modes by which bacteria cause spoilage, VCs generated in the process, and organoleptic alterations caused by spoilage.

## Factors affecting meat spoilage bacteria

### Intrinsic factors

#### pH

The pH of meat is initially 7.0 at the time of slaughter, decreasing to between 5.3 and 5.8 within 24 h after slaughter (McGeehin et al., 2001). After slaughter, interruption of blood circulation leads to anaerobic glycolysis, converting the muscle glycogen into lactic acid and decreasing pH (Onopiuk et al., 2016). As meat is stored longer, the pH is increased above 6.5 (Gill, 1983; Katiyo et al., 2020). If the pH is increased above 6.5 after slaughter, it promotes the growth of certain spoilage bacteria such as *Pseudomonas* (EFSA, 2016; Gonçalves et al., 2017). Some spoilage bacteria, *Pseudomonas* and *Serratia*, produce more active proteases at pH 6.5–7.0. (Hamamato et al., 1994; Mozhina et al., 2008; Papadopoulou et al., 2020; Patil & Chaudhari, 2011; Tsôeu et al., 2016). The proteolytic activity of spoilage bacteria may be enhanced, accelerating protein degradation and resulting in the unpleasant odor, flavor, and texture associated with spoiled meat at pH 6.5–7.0. (Joshi & Satyanarayana, 2013; Lorenzo et al., 2018).

*Pseudomonas* grew in the range of pH 5.6 to 8.0 (Urganci et al., 2022). Enterobacteriaceae grew in the range of pH 4.0 to 7.0 (Abbas et al., 2014). *Brochothrix thermosphacta* grew in the range of pH 5.0 to 9.0 (Brownlie, 1966). *Carnobacterium* grew in the range of pH 5.5 to 9.0 (Kim et al., 2009). Lactic acid bacteria (LAB) grew in the range of pH 3.0 to 8.0 (Wee et al., 2004; Vera Peña & Rodriguez Rodriguez, 2020). The pH of the food decreased to between 3.0 and 5.0 as LAB produced acetic acid or lactic acid (Mugula et al., 2003; Rhee et al., 2011). Acetic acid produced off-odor (Conte et al., 2021; Bleicher et al., 2022), and lactic acid formed a sour flavor, which may cause organoleptic alterations (Dongmo et al., 2016; Egan, 1983).

#### Water activity

Water activity (*a*_*w*_) of foods refers to a measure of water that is available for microbial growth in food, and the bacterial spoilage of food is directly affected by *a*_*w*_ (Dave & Ghaly, 2011). The *a*_*w*_ of raw meat was higher than 0.990 (Black & Jaczynski, 2008; Li et al., 2017). The high *a*_*w*_ of meat provided a favorable environment for the growth of spoilage bacteria (Ghaly et al., 2010). Most bacteria grew best at an *a*_*w*_ of 0.980–0.995 and stopped growing at *a*_*w*_<0.900 (Ghaly et al., 2010). *Pseudomonas* and Enterobacteriaceae could grow at *a*_*w*_>0.950 (Doyle & Glass, 2009). Food with low *a*_*w*_ has high storage stability. The *a*_*w*_ of meat is controlled through drying and chemical additions (e.g., salts, sugars, glycerol, and propylene glycol) (Chirife & María del Buera, 1994; Ray, 2004). In food with the *a*_*w*_ of 0.850 or less, the growth of most spoilage bacteria was inhibited (Ghaly et al., 2010).

### Extrinsic factors

#### Packaging atmosphere

The composition of spoilage flora was influenced by the packaging atmosphere (Bassey et al., 2021). *Pseudomonas*, *B. thermosphacta*, and LAB were the leading causes of spoilage in aerobically packaged meat at refrigeration temperature (Fang et al., 2022; Russo et al., 2006; Yim et al., 2019). High O_2_ concentrations, often used to maintain meat color, promoted the growth of *Pseudomonas* (Hilgarth et al., 2019).

To extend shelf-life and reduce aerobic bacterial spoilage, modified atmosphere packaging (MAP) is commonly used, which typically replaces O_2_ with carbon dioxide (CO_2_) and nitrogen (N_2_) (Conte-Junior et al., 2020; Farber, 1991; Fuertes-Perez et al., 2022). CO_2_ inhibited the metabolism and the growth of aerobic bacteria, and suppressed oxidative reactions, contributing to improved meat stability (Guo et al., 2018; Jeong & Claus, 2011; Moczkowska et al., 2017; Nauman et al., 2022). N_2_ established a low O_2_ environment that inhibited the growth of aerobic spoilage bacteria and reduced lipid oxidation (Fuertes-Perez et al., 2022; Lund et al., 2007). However, although meat was packaged in CO_2_- or N_2_-dominant MAP stored at refrigeration temperature, facultative anaerobic bacteria such as *B. thermosphacta* and LAB grew in the package (Doulgeraki et al., 2010; Hansen et al., 2023). In the case of LAB growth, the bacteria produced lactic acid, which reduced pH, and the low-pH environment inhibited the growth of low-pH-sensitive Gram-negative spoilage bacteria. It provided a competitive advantage for LAB growth (Grau, 1981; Jones et al., 2008).

#### Storage temperature

At temperatures from 0 °C to 5 °C, psychrotrophic bacteria such as *Pseudomonas*, *B. thermosphacta*, *Enterobacter* spp., and some LAB grew and caused spoilage (Nychas et al., 2008; Pennacchia et al., 2011; Rouger et al., 2017; Russo et al., 2006; Wang et al., 2017; Wickramasinghe et al., 2019; Zhang et al., 2012). In spoiled beef stored aerobically at 5 °C, *B. thermosphacta*, *Carnobacterium*, LAB and Enterobacteriaceae were detected (Mills et al., 2014; Russo et al., 2006), and *Brochothrix*, *Pseudomonas*, LAB and Enterobacteriaceae were also detected in refrigerated spoiled pork (Borch et al., 1996).

Storage temperature is an important factor in meat spoilage, as it can directly influence the growth and activity of spoilage bacteria. Storage temperature affects the enzyme activity required for bacterial growth (Berry & Foegeding, 1997; Kaur et al., 2021). Some psychrotrophic bacteria converted saturated fatty acids into unsaturated fatty acids by fatty acid desaturase, which was activated at low temperatures (Suutari & Laakso, 1994). *Pseudomonas* had Δ−9-fatty acid desaturase, which catalyzed the introduction of a double bond at the Δ−9 position of saturated fatty acyl-CoA (Choi et al., 2020). This enzyme was involved in the production of unsaturated fatty acids and played an important role in influencing the cell membrane lipid composition and regulating the cell membrane fluidity (Choi et al., 2020; Tocher et al., 1998). The cell membrane of psychrotrophic bacteria generally had a higher content of unsaturated fatty acids than that of mesophilic bacteria (Inniss, 1975; Russell, 1998; Russell & Fukunaga, 1990). Because the psychrotrophic bacteria had relatively unsaturated cell membranes, they could maintain the cell membrane fluidity even at low temperatures (Berry & Foegeding, 1997; Russell, 2002). It may allow some bacteria to grow in meat stored at refrigerated temperatures.

## Spoilage bacteria detected in spoiled meat

Table 1 lists spoilage bacteria detected in spoiled meat under different packaging conditions.

**Table 1:** Detected spoilage bacteria in meat under various storage conditions

Meat	Spoilage bacteria	Storage conditions	References	
Poultry	*Pseudomonas*	Aerobic, 4 °C		Belák et al., 2011; Mellor et al., 2011; Wang et al., 2017
	Enterobacteriaceae	Aerobic, 4 °C		Höll et al., 2016; Tsafrakidou et al., 2021
	LAB	Aerobic, 4 °C; Modified atmosphere package (70% N_2_/30% CO_2_)		
	*Brochothrix thermosphacta*	Aerobic, 8 °C; Modified atmosphere package (80% O_2_/20% CO_2_), 4–10 °C		
	*Carnobacterium*			
	*Serratia*	Aerobic, 8 °C; Modified atmosphere package (65% N_2_/35% CO_2_), 4 °C		Höll et al., 2016; Wang et al., 2017
	*Yersinia*			
Lamb	*Shewanella putrefaciens*	Vacuum-packaged, 0–5 °C		Mills et al., 2014
	*Brochothrix thermosphacta*			
	Enterobacteriaceae			
	LAB			
	*Serratia*			
	*Yersinia*			
	*Clostridium estertheticum*			
	*Clostridium gasigenes*			
	*Carnobacterium*			
Beef	*Brochothrix*	Modified atmosphere package (70% O_2_/30% CO_2_), 6 °C		Säde et al., 2017
	Enterobacteriaceae			
	*Pseudomonas*	Aerobic, 5–7 °C		Russo et al., 2006
	LAB	Aerobic, 5–7 °C		Jay et al., 2003
	*Listeria*			
	*Micrococcus*			
	*Aeromonas*			
	*Escherichia*			
	*Moraxella*			
	*Morganella*			
	*Pantoea*			
	*Providencia*			
	*Psychrobacter*			
Pork	*Pseudomonas*	Aerobic, 4 °C		Dorn-In et al., 2024
	LAB			
	*Brochothrix thermosphacta*			
	Enterobacteriaceae	Modified atmosphere package (70% N_2_/30% CO_2_), −2 °C		Bassey et al., 2021
	*Clostridium gasigenes*			
	*Brochothrix*			
	*Serratia*			
	*Carnobacterium*			
	*Acinetobacter*			
	*Rahnella aquatilis*	Vacuum-packaged, 2 °C		Godziszewska et al., 2017

In spoiled poultry, *Pseudomonas* and Enterobacteriaceae were detected under aerobic packaged conditions (Belák et al., 2011; Höll et al., 2016; Mellor et al., 2011; Tsafrakidou et al., 2021; Wang et al., 2017). LAB, *B. thermosphacta*, *Carnobacterium*, *Serratia*, and *Yersinia* were detected under both aerobic packaged and MAP (70% N_2_/30% CO_2_; 80% O_2_/20% CO_2_; 65% N_2_/35% CO_2_) conditions (Höll et al., 2016; Tsafrakidou et al., 2021; Wang et al., 2017).

In spoiled lamb, *Shewanella putrefaciens*, *B. thermosphacta*, Enterobacteriaceae, LAB, *Serratia*, *Yersinia*, *Clostridium estertheticum*, *Clostridium gasigenes*, and *Carnobacterium* were detected in vacuum-packaged conditions (Mills et al., 2014).

In spoiled beef, *Pseudomonas*, LAB, *Listeria*, *Micrococcus*, *Aeromonas*, *Escherichia*, *Moraxella*, *Morganella*, *Pantoea*, *Providencia*, and *Psychrobacter* were detected in the spoiled beef under aerobic packaged conditions (Jay et al., 2003; Russo et al., 2006). *Brochothrix* and Enterobacteriaceae were also detected under MAP (70% O_2_/30% CO_2_) conditions (Säde et al., 2017).

In spoiled pork, *Pseudomonas*, LAB, and *B. thermosphacta* were detected under aerobic packaged conditions (Dorn-In et al., 2024). Enterobacteriaceae, *C. gasigenes*, *Brochothrix*, *Serratia*, *Carnobacterium*, and *Acinetobacter* were detected under MAP (70% N_2_/30% CO_2_) conditions (Bassey et al., 2021). *Rahnella aquatilis* was also found in spoiled pork under vacuum-packaged conditions (Godziszewska et al., 2017).

These studies indicated that the detected spoilage bacteria depended on the type of meat and storage conditions, but Enterobacteriaceae, LAB, *Brochothrix*, *Serratia*, and *Carnobacterium* were generally detected in spoiled meat stored under aerobic, vacuum, and MAP conditions.

## Volatile compounds produced by meat spoilage bacteria

Meat spoilage bacteria produce compounds in the process of decomposing and metabolizing components such as proteins and fats in meat (Huang et al., 2022; Wang et al., 2018). VCs, such as VBN, VOAs, sulfur compounds, alcohols, aldehydes, and ketones in meat, are compounds that are generated as a result of microbial activity (Casaburi et al., 2015). The VCs are important indicators for evaluating the degree of spoilage and associated organoleptic alterations in meat (Mansur et al., 2019). Table 2 summarizes the modes of various VCs produced by spoilage bacteria during the decomposition of meat components.

**Table 2:** Modes of volatile compounds produced by spoilage bacteria in meat

Volatile compounds	Spoilage bacteria	Source compounds	Modes	References
Ammonia	*Pseudomonas*, some LAB	Arginine	Arginine deiminase reaction	Fröhlich-Wyder et al., 2015; Kolbeck et al., 2021; Vander Wauven et al., 1984
Amines	LAB, *Pseudomonas*, *Enterobacter*	Amino acids	Decarboxylation	Curiel et al., 2011; Li et al., 2022; Ren et al., 2022; Stadnik & Dolatowski, 2010; Zhou et al., 2022
Alcohols	LAB, Enterobacteriaceae, *B. thermosphacta*	Fatty acids, pyruvate	Enzymatic reaction (alcohol dehydrogenase, thiohydrolase, β-ketoacyl decarboxylase, reductase, etc.)	Bao et al., 2014; Bassit et al., 1993; Chen et al., 2017; Dainty & Hibbard, 1983; Dawes & Foster, 1956; Haq & Dawes, 1971; Stanborough et al., 2017; Toraya et al., 1979; Wang et al., 2022b
Aldehydes	LAB	Fatty acids	Lipid oxidation	Grebenteuch et al., 2021; McSweeney & Sousa, 2000; Pothakos et al., 2015
Ketones	LAB	Fatty acids	β-keto acid decarboxylation	Chen et al., 2017; McSweeney & Sousa, 2000; Wang et al., 2022b
Acetic acid	*B. thermosphacta*, *Carnobacterium*, Heterofermentative LAB	Glycogen	Enzymatic reaction (Phosphoketolase and/or acetate kinase)	Crans & Whitesides, 1983; Heath et al., 1958; Höll et al., 2020; Hurwitz, 1958; Novik et al., 2017; Stanborough et al., 2017; Wang et al., 2022a
Sulfur compounds	*Pseudomonas*	Methionine	Enzymatic reaction (l-methionine γ-lyase) and oxidation	Bentley & Chasteen, 2004; Esaki & Soda, 1987; Fukumoto et al., 2012; Nam & Ahn, 2003;

*Pseudomonas* and some LAB produced ammonia by the arginine deiminase reaction (Fröhlich-Wyder et al., 2015; Kolbeck et al., 2021; Vander Wauven et al., 1984). *Pseudomonas* and LAB secreted proteases that hydrolyzed proteins into peptides (Caballero et al., 2001; Savijoki et al., 2006). Free amino acids are produced from peptides through the action of peptidase (Kenny et al., 2003; Laan et al., 1998; Miller & Becker, 1978). Arginine, a free amino acid, was converted to citrulline and ammonia by arginine deiminase (Cunin et al., 1986; Rimaux et al., 2012). Citrulline was converted to ornithine and carbamoyl phosphate via ornithine transcarbamylase (Cunin et al., 1986). Carbamoyl phosphate was reacted with adenosine diphosphate by carbamate kinase to form adenosine triphosphate, and ammonia and CO_2_ were produced as final products (Cunin et al., 1986).

LAB, *Pseudomonas*, and *Enterobacter* produced amines from several different amino acids via decarboxylation (Curiel et al., 2011; Li et al., 2022; Ren et al., 2022; Stadnik & Dolatowski, 2010; Zhou et al., 2022). LAB generally have histidine decarboxylase, lysine decarboxylase, and arginine decarboxylase (Barbieri et al., 2019). *Pseudomonas* has lysine decarboxylase and arginine decarboxylase, and *Enterobacter* has histidine decarboxylase and lysine decarboxylase (Komprda et al., 2010; Pircher et al., 2007; Ren et al., 2022; Rosenfeld & Roberts, 1976; Santos, 1998). Histidine decarboxylase, lysine decarboxylase, and arginine decarboxylase processed histidine, lysine, and arginine to histamine, cadaverine, and putrescine, respectively (Lucas et al., 2005; Pircher et al., 2007).

The lipase possessed in LAB broke down triglycerides in meat into fatty acids (Tanasupawat et al., 2015; Silva Lopes et al., 1999). In a study by Chen et al. (2017), aldehydes were produced from unsaturated fatty acids through the action of hydroperoxide lyase, and aldehydes were reduced to alcohols. Methyl ketone was produced from saturated fatty acids through the action of thiohydrolase and β-ketoacyl decarboxylase, and methyl ketone was reduced by reductase to secondary alcohols such as 2-propanol and 2-butanol (Chen et al., 2017; Wang et al., 2022b). Enterobacteriaceae produced ethanol from pyruvate via pyruvate decarboxylase and alcohol dehydrogenase (Dawes & Foster, 1956; Haq & Dawes, 1971; Toraya et al., 1979). Pyruvate was converted to α-acetolactate by α-acetolactate synthase in *B. thermosphacta* (Dainty & Hibbard, 1983; Stanborough et al., 2017). The α-acetolactate was converted to diacetyl and CO_2_ by oxidation, and the diacetyl was reduced to acetoin by diacetyl reductase (Bassit et al., 1993). Acetoin was reduced to the secondary alcohol, 2,3-butanediol, by butanediol dehydrogenase (Bao et al., 2014; Kersters & De Ley, 1963).

Aldehydes are organic compounds containing a carbonyl group (C = O) and characterized by a distinctive odor (Attaway et al., 1962; El Hadi et al., 2013). LAB produced the aldehydes (Pothakos et al., 2015). LAB promoted lipid oxidation in meat by lipoxygenases (McSweeney & Sousa, 2000). Oxygen reacts with fatty acids to produce primary oxidation products, which are hydroperoxides, and further oxidation produces aldehydes such as hexanal, heptanal, and octanal (Grebenteuch et al., 2021).

Ketones are secondary products formed during lipid oxidation and bacterial dehydrogenation of secondary alcohols (Casaburi et al., 2015). The presence of ketones was associated mainly with LAB (Li et al., 2018). LAB produced keto acyl-CoA by β-oxidation of saturated fatty acids (Wang et al., 2022b). The keto acyl-CoA was converted to β-keto acids by thiohydrolase and to methyl ketone by β-ketoacyl decarboxylase (Chen et al., 2017; McSweeney & Sousa, 2000).

Muscle glycogen in meat was broken down into glucose with several enzymatic reactions (Bai et al., 2020). Hexokinase converted glucose to glucose-6-phosphate (Hass et al., 1961). Heterofermentative LAB converted glucose-6-phosphate to 6-phospho-gluconate by glucose-6-phosphate dehydrogenase (DeMoss et al., 1953; Kandler, 1983). The 6-phospho-gluconate was converted to xylulose-5-phosphate through a metabolic process by 6-phospho-gluconate dehydrogenase and ribulose-5-phosphate-3-epimerase (Gumaa & McLean, 1969; Novello & McLean, 1968; Novik et al., 2017). The xylulose-5-phosphate was then converted to acetyl phosphate by phosphoketolase present in heterofermentative LAB (Heath et al., 1958; Hurwitz, 1958; Novik et al., 2017; Wang et al., 2022a). Acetyl phosphate was converted to acetic acid by acetate kinase in heterofermentative LAB (Crans & Whitesides, 1983). Additionally, *B. thermosphacta* and *Carnobacterium* known to be involved in meat spoilage (Casaburi et al., 2015), also produce acetic acid through acetate kinase activity, thereby lowering the quality of meat (Höll et al., 2020; Stanborough et al., 2017).

Some meat proteins contained sulfur-containing amino acids such as methionine (Schutte & Teranishi, 1974). *Pseudomonas* produced sulfur compounds (Pohl et al., 1984). *Pseudomonas* possessed l-methionine γ-lyase, which converted methionine into methanethiol (Fukumoto et al., 2012). Afterward, methanethiol was converted to odor-causing dimethyl disulfide by oxidation (Bentley & Chasteen, 2004; Esaki & Soda, 1987; Nam & Ahn, 2003).

## Organoleptic alterations due to meat spoilage

### Slime formation

In the early stages of spoilage, bacterial growth caused slime formation on the surface of meat (Cenci-Goga et al., 2020). The slime was formed by the bacterial exopolysaccharides (EPS) (Iulietto et al., 2015). Different species of LAB, such as *Leuconostoc*, *Lactobacillus sakei*, *Lactobacillus curvatus*, *Lactobacillus helveticus*, *Lactobacillus casei*, *Lactobacillus fermentum*, and *Lactobacillus plantarum*, synthesized various EPS (Jurášková et al., 2022; Ray & Bhunia, 2013). The nomenclatures of *L. sakei*, *L. curvatus*, *L. casei*, *L. fermentum*, and *L. plantarum* were changed to *Lactilactobacillus sakei*, *Lactilactobacillus curvatus*, *Lacticaseibacillus casei*, *Limosilactobacillus fermentum*, and *Lactiplantibacillus plantarum*, respectively (ISAPP, 2020; Zheng et al., 2020). EPS synthesis in LAB was initiated by the activation of sugar nucleotides such as UDP-glucose and UDP-galactose, and the synthesis was progressed by the assembly of repeating sugar units through enzymatic reactions (Liu et al., 2025; Ramos et al., 2001).

Slime can also be formed by protein decomposition, which was facilitated by enzymes secreted by bacteria during meat storage (Wickramasinghe et al., 2019). *Pseudomonas* secreted proteases with vigorous proteolytic activity against proteins found in myofibrillar and myomatrix proteins (Ołdak & Trafny, 2005; Pellissery et al., 2020; Stanborough et al., 2018). The amino acids were produced by protein degradation accumulated on the surface of the meat, eventually forming a slimy layer (Katiyo et al., 2020).

### Odor

Odor is an organoleptic characteristic resulting from the production of VCs (Wood et al., 2004). *Pseudomonas* are a type of bacteria that can break down meat protein more efficiently than non-proteolytic bacteria such as *Brochothrix* (Koutsoumanis et al., 2005). This was because *Pseudomonas* secreted a digestive enzyme that can break down the connective tissue between muscle fibers (Koutsoumanis et al., 2005). As the proteins were broken down, sulfur compounds such as methanethiol (CH_3_SH) and dimethyl sulfide (C_2_H_6_S) were found in meat spoiled by *Pseudomonas* (Stanborough et al., 2018). The sulfur compounds have an off-odor similar to rotten eggs or cabbage (Bekker et al., 2016).

Branched-chain amino acids (BCAAs), such as leucine, isoleucine, and valine, are commonly found in meat protein (Kim et al., 2022). *Pseudomonas* produced branched-chain keto acids (BCKAs), such as ketoleucine, ketoisoleucine, and ketovaline via aminotransferase-catalyzed transamination of BCAAs (Dimou et al., 2022; Fulton & Downs, 2024; Martin et al., 1973). These BCKAs could then be further converted to branched-chain fatty acid (BCFA) through decarboxylation in *Pseudomonas* (Sokatch et al., 1981). These decarboxylation processes lead to fatty acid formation, causing an unpleasant odor in meat (Carballo, 2012).

LAB promoted the oxidation of fatty acids by lipoxygenase to produce aldehydes with a rotten, fruity odor (Curren et al., 2016; McSweeney & Sousa, 2000). LAB secreted lipase to hydrolyze lipids into glycerol and fatty acids in meat (Guan et al., 2020; Tanasupawat et al., 2015). The LAB reduced aldehydes produced from fatty acids to alcohols (Chen et al., 2017). LAB oxidized fatty acids by thiohydrolase and β-ketoacyl decarboxylase to produce ketones that smell like acetone (Chen et al., 2017; McSweeney & Sousa, 2000).

Heterofermentative LAB have phosphoketolase and acetate kinase, which can produce acetic acid from glycogen (Crans & Whitesides, 1983; Novik et al., 2017; Wang et al., 2022a) and *B.* *thermosphacta* and *Carnobacterium* also have acetate kinase, which allows them to produce acetic acid in meat (Höll et al., 2020; Stanborough et al., 2017). Acetic acid causes a pungent, acidic, and cheesy off-odor in spoiled meat (Mansur et al., 2019; Pothakos et al., 2015).

The mode for producing odor-causing VCs by decomposing lipids by spoilage bacteria was explained in Sect. Volatile compounds produced by meat spoilage bacteria.

### Color

Color changes in meat occurred due to bacteria on the surface when the bacterial population was about 10^8^ CFU/cm^2^ (Brooks et al., 2008; Chan et al., 1998). *Pseudomonas* produced hydrogen sulfide, which is responsible for the change to green color (Borch et al., 1996). The hydrogen sulfide was bound to the iron atom in the heme group of myoglobin (Cirino et al., 2023; Pietri et al., 2011). This reaction formed sulfmyoglobin (Nicol et al., 1970; Román-Morales et al., 2016), which affected the electron distribution around the iron atom (Pietri et al., 2009). This alteration in electron distribution changed the light absorption properties of the iron atom in the heme group, shifting the absorbance wavelengths that showed red to those that reflected green (Libardi et al., 2013). Consequently, the formation of sulfmyoglobin resulted in a color change from the normal red of myoglobin to a green color (Brewer, 2004).

## Conclusions

The spoilage bacteria depend on the type of meat and storage conditions (packaging atmosphere, temperatures, pH, *a*_*w*_, etc.), *Pseudomonas*, LAB, Enterobacteriaceae, *B. thermosphacta*, and *Carnobacterium* are general spoilage bacteria that may cause meat spoilage. *Pseudomonas* produces amines, ammonia, and sulfur compounds from proteins. LAB produce acetic acid from glycogen, amines and ammonia from proteins, and alcohols, aldehydes and ketones from lipids. Enterobacteriaceae decompose glycogen to produce ethanol, and proteins to produce amines. *B. thermosphacta* produces alcohols and acetic acid from glycogen. *Carnobacterium* breaks down glycogen to produce acetic acid. These VCs produced by spoilage bacteria may generally contribute to organoleptic alterations. The information reviewed in this paper should help understand meat spoilage by bacteria. However, this paper primarily addresses meat spoilage only by bacteria, and thus, other causes responsible for spoilage are not reviewed in this paper. In addition, the information from limited or inconclusive research data was not discussed in detail. Therefore, further studies are necessary to supplement these insufficient research results for the bacterial spoilage in meat.
